# Minimally invasive detection of early-stage opisthorchiasis-associated cholangiocarcinoma using label-free surface-enhanced Raman spectroscopy (SERS) of hamster serum

**DOI:** 10.1371/journal.pone.0334916

**Published:** 2025-10-27

**Authors:** Apisit Chaidee, Suppakrit Kongsintaweesuk, Thatsanapong Pongking, Keerapach Tunbenjasiri, Aye Myat Mon, Chawalit Pairojkul, Pakornkiat Tanasuka, Tullayakorn Plengsuriyakarn, Kesara Na-Bangchang, Naruechar Charoenram, David Blair, Somchai Pinlaor

**Affiliations:** 1 Department of Parasitology, Faculty of Medicine, Khon Kaen University, Khon Kaen, Thailand; 2 Cholangiocarcinoma Research Institute, Faculty of Medicine, Khon Kaen University, Khon Kaen, Thailand; 3 Faculty of Associated Medical Sciences, Centre for Research and Development of Medical Diagnostic Laboratories, Khon Kaen University, Khon Kaen, Thailand; 4 Biomedical Sciences Program, Graduate School, Khon Kaen University, Khon Kaen, Thailand; 5 Department of Pathology, Faculty of Medicine, Khon Kaen University, Khon Kaen, Thailand; 6 Graduate Program in Bioclinical Sciences, Chulabhorn International College of Medicine, Thammasat University (Rangsit Campus), Pathumthani, Thailand; 7 Center of Excellence in Pharmacology and Molecular Biology of Malaria and Cholangiocarcinoma, Thammasat University (Rangsit Campus), Pathumthani, Thailand; 8 College of Science and Engineering, James Cook University, Townsville, Queensland, Australia; Institute of Cytology and Genetics SB RAS: FIC Institut citologii i genetiki Sibirskogo otdelenia Rossijskoj akademii nauk, RUSSIAN FEDERATION

## Abstract

**Background:**

Cholangiocarcinoma (CCA) is a deadly cancer often detected late. Current diagnostic methods, such as ultrasound and invasive biopsies, have limitations; there is a critical need for a rapid, minimally invasive and effective strategy for the early diagnosis and staging of CCA.

**Methods:**

We aimed to address this need using serum samples and label-free surface-enhanced Raman spectroscopy (SERS) combined with machine learning. CCA development was induced in hamsters using a combination of *Opisthorchis viverrini* infection and administration of *N*-nitrosodimethylamine, with induction time courses spanning 1–5 month(s). Normal and pathological stages (inflammation, precancerous lesion, and CCA) were assigned based on histopathological features, as well as the expression of cytokeratin 19 and alpha-fetoprotein. Raman spectra were subjected to dimensionality reduction using principal component analysis, and diagnostic clusters were acquired using partial least-squares discriminant analysis.

**Results:**

Histopathological analysis confirmed a clear path towards CCA, initiated by marked inflammation, progressing to include significant cholangiofibrosis and cholangiofibroma in the precancerous stage, and culminating in definitive CCA tumor development. The integration of SERS and machine learning achieved a diagnostic sensitivity of 93%, specificity of 95%, and accuracy of ≥ 67% for precancerous lesions and CCA, with an area under the receiver operating characteristic curve exceeding 0.67.

**Conclusions:**

Our findings demonstrate that this cost-effective, label-free SERS approach can accurately detect precancerous and cancerous stages of cholangiocarcinoma in a hamster model, highlighting its strong potential for future development as a community-based screening tool.

## Introduction

Cholangiocarcinoma (CCA), a malignancy arising from the biliary epithelial cells of the intrahepatic, perihilar, and distal biliary trees, is a growing global health challenge [1]. While relatively rare, its incidence has been steadily increasing over recent decades, with significant regional disparities [2]. Globally, CCA exhibits an incidence and mortality rate of 0.3–6 cases per 100,000 people annually, but in hotspot regions such as South Korea, China, and Thailand, rates exceed 6 per 100,000 [3]. Infection with small liver flukes, such as *Opisthorchis viverrini* (OV), is a recognized risk factor for CCA in the greater Mekong subregion, encompassing East and Southeast Asia [4]. Similarly, the foodborne trematode *Clonorchis sinensis* causes CCA through chronic infection of the bile ducts, making the disease prevalent in parts of China and Korea [5]. In contrast, parasite-associated CCA remains rare in Western countries, a difference primarily attributed to cultural and dietary habits that limit the consumption of raw or undercooked freshwater fish [6].

A critical obstacle in CCA management is the difficulty of early detection. Due to the often-vague symptomatology and the lack of robust, widely available screening tools and biomarkers, particularly for non-high-risk individuals, diagnosis frequently occurs at advanced stages, hindering effective intervention. Current reliance on imaging techniques alone proves insufficient for accurate, cost-effective, and timely diagnosis. Thus, there is an urgent need for tools to facilitate the timely detection of CCA [7]. Previous research in a hamster CCA model utilising spectroscopy-based techniques, such as synchrotron radiation-FTIR (SR-FTIR) microspectroscopy and focal-plane array-FTIR (FPA-FTIR) microspectroscopy, has successfully distinguished bile-duct neoplasms across various disease stages, from uninfected to malignant [8]. However, these methods require expensive instruments, trained operators and are not suitable for use in typical primary healthcare settings.

In recent years, Raman spectroscopy has emerged as a powerful tool for cancer assessment, diagnosis, and perioperative surgical guidance, owing to its chemical precision and non-invasive monitoring capabilities [9]. Raman confocal microscopy detects molecular vibrations that are specific to particular biomolecules to produce biochemical information, in contrast to traditional confocal microscopy, which primarily offers morphological information through fluorescence or reflection [10]. This technique offers a versatile approach for label-free diagnosis, including the detection of various cancers, such as CCA [11], liver [12], breast [13], and lung cancer [14], via serum analysis. Combining Raman spectroscopy with machine learning enhances the ability of this approach to distinguish subtle spectral variations between healthy and diseased groups. However, the inherently limited cross-sectional area of Raman scattering from biomolecules can pose a challenge in detecting early-stage cancers with minor biochemical changes. To address this, surface-enhanced Raman spectroscopy (SERS) can amplify the Raman signal, boosting diagnostic accuracy, particularly for early CCA detection [11]. Surface-enhanced Raman scattering (SERS), with its heightened sensitivity and multiplexing capabilities, holds great promise for biological analysis and diagnostics [15]. Moreover, it should be possible to eventually develop a portable device that makes SERS available as a point-of-care test and for community utilization [16,17].

This study aims to establish a rapid, serum-based, label-free surface-enhanced Raman spectroscopy (SERS) technique coupled with machine learning to detect the early stages of CCA in hamsters, where the cancer was induced using a combination of *O. viverrini* infection and administration of *N*-nitrosodimethylamine (NDMA). The findings of this study introduce novel strategies not only for the early diagnosis of CCA but also for developing point-of-care CCA screening, which could potentially lead to an effective reduction in CCA mortality and management costs.

## Materials and methods

### Animals used and ethical statement

A total of 60 male Syrian golden hamsters (*Mesocricetus auratus*), aged 4–6 weeks and weighing between 80 and 100 g, were selected at random for the experiment. The hamsters were housed at the Animal Unit of the Faculty of Medicine, Khon Kaen University, Thailand, for a minimum of 5 days before the start of the experiment. Hamsters were group-housed (5 animals per cage) to prevent social isolation stress, a potential confounding factor in studies of inflammation and cancer. To mitigate the risk of aggression, all cages were provided with environmental enrichment and were monitored daily, with established protocols for separating animals if necessary. No animals were required to be separated during the study. The temperature was maintained at 23°C with a variation of ± 2°C. The relative humidity ranged from 30% to 60%, and a 12-hour cycle of light and darkness was maintained. The hamsters were given a commercial pellet meal (CP-SWT, Thailand) freely and were supplied with water ad libitum. The stainless-steel cages were cleaned weekly using detergent, disinfected with the antimicrobial agent Dettol (Dettol, Thailand) to prevent bacterial infection, and the sawdust was replaced twice a week. The behavior and general health of the hamsters were monitored daily. Throughout the experiment, most hamsters did not meet the predetermined criteria for humane euthanasia endpoints, such as significant weight loss, changes in appetite, dehydration, adverse behavioral changes, or other clinical signs of distress. However, a few hamsters exhibited severe pain and aggression due to inter-animal biting, evidenced by multiple body wounds, which unfortunately led to their death the following day. Consequently, to prevent further suffering, the remaining hamsters were euthanized prior to the study’s scheduled endpoint. All animal care procedures, including husbandry and euthanasia, were performed by certified and competent staff in strict adherence to ethical guidelines.

The study’s protocol (IACUC-KKU-56/66) was assessed and authorized by the Animal Ethics Committee of Khon Kaen University, following the ethical criteria for animal testing established by the National Research Council of Thailand. All animal procedures were conducted in compliance with the NIH Guide for the Care and Use of Laboratory Animals (https://grants.nih.gov/grants/olaw/guide-for-the-care-and-use-of-laboratory-animals.pdf), ensuring adherence to internationally recognized ethical standards. The animal study was conducted in accordance with the Animal Research: Reporting of In Vivo Experiments (ARRIVE) guidelines [18], ensuring transparent and rigorous reporting of all experimental procedures.

### Experimental design

Sixty hamsters were randomly assigned to two groups: a normal control group (n = 15, from which 5 animals were sacrificed at each of 1, 3, and 5 months) and a group infected with OV and provided with NDMA (n = 45). Those in the second group were sampled at 1 (n = 5), 3 (n = 5), 4 (n = 12), and 5 (n = 23) months after the start of CCA induction. The purpose of the different sampling times was to provide lesions at different stages. The different animal numbers at 4 and 5 months reflect mortalities before scheduled euthanasia. To increase the sample size of precancerous and cancerous samples, leftover samples from 4 and 5 months in a previous study [19] were also recruited.

Metacercariae of OV were isolated and quantified after digestion of naturally infected cyprinid fish in a solution containing 0.25% pepsin and 1.5% hydrochloric acid (Wako Pure Chemical Industries, Osaka, Japan) in a 0.85% sodium chloride solution. Each hamster received a total of fifty live OV metacercariae by intragastric gavage. NDMA was administered in drinking water at a concentration of 12.5 parts per million (ppm) for one month and then withdrawn.

After 5 months of the experiment, all the hamsters were starved of food for a day before being killed. Each hamster was anesthetized and euthanized by isoflurane inhalation. Following a cardiac puncture, blood was collected into a serum-separator tube. The tube was centrifuged for ten minutes at 4°C and 3500 rpm. Before being used for Raman spectroscopy analysis, serum was separated, and aliquots were stored at −80°C until analysis.

### Hematoxylin and eosin (H&E) staining

Liver tissues embedded in paraffin wax were sectioned (4 µm) and processed using a standard hematoxylin and eosin (H&E) staining protocol, as previously published elsewhere. The definition and scoring of histopathological changes, including active inflammation, cholangiofibrosis, cholangiofibroma, and cholangiocarcinoma lesions, were described as previously reported [19–21]. Three independent investigators performed blinded evaluations, which were then confirmed by a senior pathologist.

### Immunohistochemical staining

Immunohistochemistry was performed on liver sections (4 μm thickness) from all experimental groups. The paraffin-embedded tissue slices were deparaffinised using three changes of xylene, followed by rehydration in 100%, 95%, and 70% ethyl alcohol, and finally in water for 5 minutes in each change. Then, the antigen was retrieved from the tissue by autoclaving in a sodium citrate buffer (10 mM sodium citrate, 0.05% Tween 20, and pH 6.0). The tissue sections were submerged in 3% H_2_O_2_ for 10 minutes and incubated with 5% BSA for one hour at room temperature. The antibodies used were rabbit anti-CK19 (1:100 dilution, ab15463, Abcam, UK) and rabbit anti-AFP (1:500 dilution, GA500, Dako Omnis, USA), diluted in 1% fetal bovine serum (FBS). The tissue sections were incubated with antibodies at 4°C overnight in a humidified environment. Only the antibody diluent was administered to the control sections to confirm the antibody’s specificity. On the following day, the PBS-washed microscope slides were left to stand at room temperature for one hour while goat anti-rabbit IgG, a secondary antibody (1:200 dilution, 515-035-003, Jackson immunoresearch, PA, USA), was applied in a 5% BSA solution. Subsequently, slides were stained with Mayer’s hematoxylin after 3,3-diaminobenzidine was employed to activate antibody reactions. The slides were mounted using Permount (Thermo Fisher Scientific, MA, USA) and coverslips. The staining intensity was evaluated using an ordinary light microscope and analyzed using ImageJ (National Institutes of Health, MD, USA).

### Stage-specific CCA development

Cholangiocarcinoma stages in the animal model were categorized into four levels: normal tissue, inflammatory lesion, pre-cancerous lesion, and tumor. Inflammatory lesions, identified by the accumulation of inflammatory cells and fibrosis, were observed in all samples from the OV+NDMA group one month after the start of treatment. Precancerous lesions, defined by the presence of inflammation, fibrosis, cholangiofibrosis, and cholangiofibroma, were predominantly identified within three months. Cancerous lesions, characterized by the presence of inflammation, fibrosis, cholangiofibrosis, cholangiofibroma, and tumorous tissue, were primarily observed at four and five months, with some cases also noted at three months. Cholangiofibrosis, cholangiofibroma, and tumorous lesions were confirmed by both histopathological features and positive immunohistochemical staining of tumor markers. For bile duct cancer, this was cytokeratin 19 (CK19), and for liver cancer, alpha-fetoprotein (AFP) was used. CK-19 is a valuable biomarker, not only for the early detection of CCA but also for its crucial differentiation from hepatocellular carcinoma (HCC) [22]. Conversely, AFP, a glycoprotein typically elevated in HCC, serves as a negative marker for CCA, where it shows only occasional nonspecific increases [23]. The use of this biomarker profile has global diagnostic recognition, as it can confirm a CCA diagnosis regardless of its parasitic or non-parasitic etiology.

Chronic active inflammation was defined as a mixture of acute and chronic inflammatory cells in the periductal tissue, surrounding liver tissue, and bile ducts [24]. Cholangitis severity was scored independently for perihilar and peripheral bile ducts using a six-point scale: 0 (no cholangitis) to 5 (severe cholangitis), with intermediate grades representing marginal, slight, moderate, and marked inflammation [24]. Both the cholangitis grading and pathological diagnoses were conducted by a minimum consensus of two independent researchers. Furthermore, a senior pathologist confirmed the results, as previously reported [19].

### Preparation of film-based Ag-nanorod SERS sensors (ONSPEC Prime)

Nanostructured silver films, fabricated via physical vapor deposition, served as surface-enhanced Raman scattering (SERS) substrates as previously described [25]. These films were synthesized using a combined magnetron sputtering and glancing-angle deposition method, which involves continuous substrate tilting during deposition. Specifically, the substrate holder was inclined at 85 degrees relative to the normal plane of the sputtering cathode and simultaneously rotated at 5 revolutions per minute. A 99.99% pure silver target (K.J. Lesker) was sputtered onto p-type silicon wafers. The morphology of the resulting nanostructures was characterized using a Hitachi SU8030 field-emission scanning electron microscope. For SERS analysis, the substrates were diced into 5.0 mm × 5.0 mm chips and mounted onto handling materials. Six independent fabrication runs, employing identical procedures, were conducted to produce film-based surface-enhanced Raman scattering (SERS) sensors. Randomly selected sensors from each run were utilized for SERS analysis of hamster serum. To minimize environmental effects, sensors were stored in nitrogen-filled, metalized bags and used within one week of fabrication.

### Raman spectrum detection based on the SERS sensors

Our SERS-based detection differed somewhat from a previously reported method [26]. The SERS experiments were carried out using the XploRA Plus (Horiba, Kyoto, Japan) confocal Raman microscope, a state-of-the-art device manufactured by Horiba Jobin Yvon (UK). Each hamster’s serum was individually applied to the center of the silver-based SERS sensors in an amount of 2.0 μl, and the samples were allowed to air-dry for ten minutes. A Raman spectrum was obtained by analyzing the central area of every SERS sensor using a laser with a wavelength of 785 nm and a 50 × objective lens. Each spectrum capture was performed with a laser intensity of 25 milliwatts and an exposure time of 10 seconds. For every sample, 49 points, comprising a 7 × 7 grid with a step size of 15 μm, were used to collect the spectral data.

### Optimization of Raman spectrum measurement

To determine the optimal serum dilution for surface-enhanced Raman spectroscopy (SERS) analysis, serum samples were diluted with deionized water in a two-fold serial dilution series, ranging from 1:20–1:320. Each sample was measured at 49 points, and signal data were obtained for 40 points at a 1:80 dilution, 30 points at a 1:160 dilution, and fewer than 24 points at a 1:320 dilution. The dilution series was prepared to evaluate the influence of sample concentration on SERS signal intensity and spectral quality. Raman spectra were acquired using an XploRA Plus Raman spectrometer. A 785 nm laser was used as the excitation source, with an acquisition time of 10 seconds per spectrum and a single accumulation. The spectrometer settings included a 1200 grating (750 nm) and a 25% filter. Spectra were collected using a 50 × objective lens. The Raman spectral range was set from 400 to 1800 cm ⁻ ¹ based on a preliminary study. The Raman spectral data from different serum dilutions were analyzed to determine the optimal dilution factor for achieving high spectral quality and reproducibility. Signal intensity, baseline noise, and spectral resolution were evaluated to determine the dilution that yielded the optimal SERS enhancement with minimal background interference. After obtaining the Raman spectra, we analyzed the data to select the optimum spectra for further analysis.

### Statistical assessment and machine-learning approach for analysis of Raman spectrum data

We employed principal component analysis (PCA) to reduce the dimensionality of the data and extract related signals from the otherwise statistical variables, thereby improving the identification of important peaks and shifts in the Raman spectra. The entire spectrum is covered by the 820 spectral intensity data in the input vector variable, which ranges from 450 to 2250 cm^−1^ in 2 cm^−1^ increments. To find the average of all the Raman spectra from each group, we plotted the standard deviation from the group average spectrum as error bars. The Raman data was plotted on the original PCs to differentiate it based on its cross-product with the principal components (PCs) visually.

To further distinguish between the control and infected groups based on subtle spectrum changes, we employed linear discriminant analysis (LDA.). We eliminated redundant spectral features using LDA, which increased classification accuracy and model efficiency. Next, we plotted LDA to demonstrate the degree of separation between various grouped samples in the condensed space. To assess classification models in SERS, we also employed a train-test split and leave-one-out-cross-validation (LOOCV) to see how well they generalize to new spectral data. In order to accurately identify even the smallest spectral differences, we further verified using LOOCV that each SERS spectrum could be evaluated independently. All data processing and machine-learning models were implemented in Python (v3.10.11) using the scientific computing libraries NumPy, pandas, and scikit-learn (v1.0.2).

Raman mapping of 50 hamster specimens yielded 49 spectra per sample, creating a dataset of 2,450 spectra for analysis. Given the hierarchical nature of this data (multiple spectra nested within each animal), we employed two distinct validation strategies to train our four-class supervised learning models. First, as a baseline, we performed a random spectrum-level split (2/3 training, 1/3 test) to assess the model’s fundamental discriminatory power across the entire dataset. Second, to ensure model generalizability and prevent data leakage, we implemented a more rigorous LOOCV. In this primary approach, all spectra from a single hamster were used for validation while the model was trained on spectra from the remaining 49 animals. This process was repeated until every specimen had served as the validation set, guaranteeing that spectra from the same animal were never present in both training and validation sets. This sample-level validation provides a robust and unbiased estimate of performance, mitigating the risk of accuracy overestimation as highlighted in recent literature [27,28].

## Results

### Stage scoring of CCA development in the hamster model

Liver tissue from each hamster was stained using H&E and then further subjected to immunohistochemistry for tumor markers. Table 1 presents the distribution of animals assigned to histopathological stages of CCA development, categorized by duration of CCA induction. In the OV+NDMA group, four animals exhibiting only fibrotic lesions (one 4-month-old, and three 5-month-old) and lacking both parasitic infection and neoplastic lesions were excluded from this study. Six animals in the same group that died before scheduled euthanasia at 4 and 5 months were also excluded. Consequently, fifty animals contributed data for further analysis, as summarized in Table 1.

**Table 1:** Histopathology-based number of hamsters treated with OV and NDMA and associated lesion stages.

Time of induction	Normal	Inflammation	Pre-cancer	CCA
1 month	5	5	–	–
3 months	5	–	3	2
4 months	–	–	5	4
5 months	5	–	7	9

**Fig 1:**
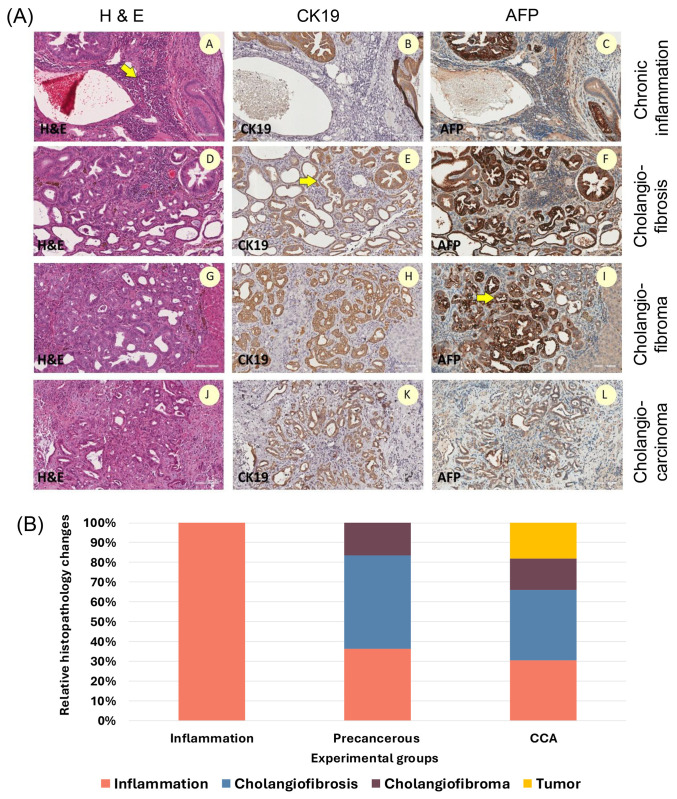
Illustrative micrographs and comparative analysis in each stage of CCA development. CCA and its precursor lesions in sections stained with hematoxylin and eosin (H&E) and with cytokeratin 19 (CK19), and alpha-fetoprotein (AFP) immunohistochemical stains. **(A)** The stages of CCA progression in hamster livers infected with OV and receiving NDMA. A–C Transformation of chronic inflammation, D-F malignant transformation of cholangiofibrosis; G-I malignant transformation of cholangiofibroma; J-L CCA, a well-differentiated adenocarcinoma producing mucin; malignant transformation from a dilated metaplastic duct. **(B)** Comparative analysis of histopathological findings in three different stages of CCA development. Raw data of histopathological findings by the investigators are shown in S1 Table and S2 Table. Yellow arrowheads indicate abnormalities in bile ducts.

Fig 1A (A-C) illustrates chronic OV infection-induced cholestasis, which results in healed cholangitis and active inflammation that may sometimes progress to cholangiofibrosis. The latter manifests as small, grayish granules inside the parenchyma. Cholangiofibrosis lesions consisted of foci of intestinal metaplasia, hyperplastic bile ductules, and fibrotic stroma as shown in Fig 1A **(D-F)**. Cholangiofibroma is a condition characterized by the enlargement of nodules in the liver, which appear as gray, well-defined lesions. These nodules may exert pressure on the surrounding liver tissue as shown in Fig 1A(G-I). CCA is a focused, grayish-white liver tumor that might appear as a cluster of plaques or nodules. The adenocarcinoma consists of glands, solid sheets, trabeculae, or tightly packed ductules. It may or may not produce mucus as shown in Fig 1A **(J-L)**. More tumor mass, cholangiofibroma, and active inflammation were seen at 5 months post-treatment. Cholangiofibrosis, cholangiofibroma, and adenocarcinoma were stained for both CK19−9 and AFP expression, confirming the presence of pre-cancerous and cancerous lesions. The histopathological findings in all the experimental groups are shown in Fig 1A.

The proportion of each grade scored as inflammation, cholangiofibrosis, cholangiofibroma, or tumor is presented in Fig 1B. Only the inflammatory condition was seen after 1 month in the OV+NDMA group. Sections classified as precancerous exhibited, in sequential order of development or co-occurrence, cholangiofibrosis, active inflammation, and cholangiofibroma. Within cancerous tumor lesions, the findings included cholangiofibrosis, active inflammation, tumor formation, and cholangiofibroma.

### Histopathological progression of CCA

Stage-specific alterations were revealed by histopathological evaluation (Table 2, S1Table and S2 Table). There were a few additional lesions and a high level of inflammation (mean = 1.8) in the inflammation group. The precancerous group demonstrated increased inflammation (2.23), marked cholangiofibrosis (3.03), and cholangiofibroma (1.07) without tumors in mean values. In the CCA group with mean values, inflammation (2.13), cholangiofibrosis (2.47), and cholangiofibroma (1.1) persisted with tumor formation (1.27). These findings suggest a pathological sequence linking inflammation, cholangiofibrosis, and cholangiofibroma to CCA development.

**Table 2:** Histopathological findings in hamster livers of all the experimental (OV+NDMA) groups.

Histopathological staging	Persistent Inflammation		Cholangiofibrosis		Cholangiofibroma		Tumor development	
Mean	Percentage	Mean	Percentage	Mean	Percentage	Mean	Percentage
Inflammation (n = 5)	1.8	100%	0.0	0%	0.0	0%	0.0	0%
Precancerous (n = 15)	2.23	36%	3.03	47%	1.07	17%	0.0	0%
CCA (n = 15)	2.13	31%	2.47	35%	1.1	16%	1.27	18%

### Optimization of serum samples for surface-enhanced Raman spectroscopy

**Fig 2:**
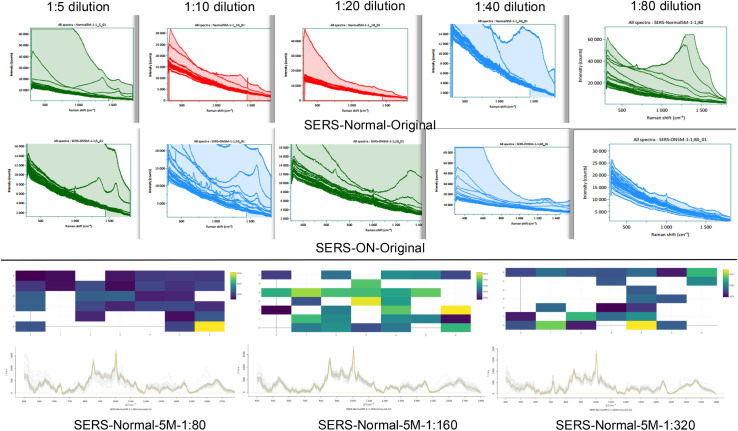
Optimization of serum dilution (using deionized water) for Raman spectroscopy. Signal data were acquired from 40 points per chip at the lower dilutions of 1:5 to 1:40, at 37 points for the 1:80 dilution, 29 points for the 1:160 dilution, and fewer than 24 points at the 1:320 dilution.

We diluted serum samples with deionized water (from 1:5–1:320) to determine the optimum dilution factor for Raman spectroscopy (Fig 2). The results indicated that lower dilution factors (1:5–1:40) resulted in high background and unclear Raman peaks. The 1:80 dilution exhibited the most consistent Raman signals with clear peak resolution and optimal SERS enhancement. At 1:160, spectral quality improved, but some baseline fluctuations remained, which could interfere with spectral feature extraction. The 1:320 dilution provided the lowest Raman signals, and background noise remained. Based on these findings, the 1:80 dilution was selected for further analysis, as it provided a balance between signal intensity and spectral clarity, ensuring reliable peak identification and machine-learning classification.

### Surface-enhanced Raman acquisition and peak assignment for each group

**Fig 3:**
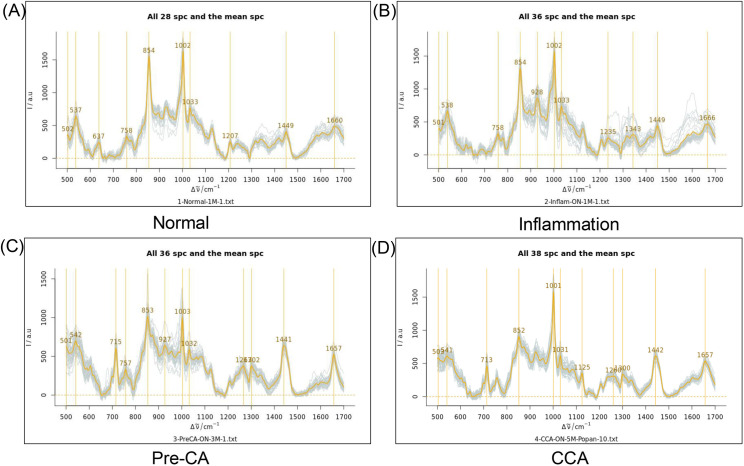
Representative spectral peaks of SERS in different stages. Indicative SERS based on four different pathology categories: (A) normal, (B) inflammation, (C) precancerous (Pre-CA), and **(D)** CCA.

Raw data from Raman spectroscopy were not processed in the same way as previously reported [26]. The comparison of Raman spectral peaks among the four pathology categories (normal, inflammation, precancerous (Pre-CA), and CCA) revealed distinct biochemical differences associated with CCA progression. As shown in Fig 3, each group exhibited characteristic spectral features, with key Raman peaks observed at multiple wavenumbers. The normal group showed well-defined peaks at 537, 637, 758, 854, 1002, 1449, and 1660 cm ⁻ ¹, indicative of baseline biochemical composition, as shown in Fig 3A. The inflammation group exhibited notable intensity shifts, particularly at 538, 758, 854, 1002, 1449, and 1666 cm ⁻ ¹, suggesting metabolic alterations related to inflammatory responses (3B). The Pre-CA group displayed significant changes in peak intensity, with shifts observed at 542, 715, 1003, 1441, and 1657 cm ⁻ ¹, reflecting early-stage cancer-associated metabolic disruptions, as presented in Fig 3C. In the CCA group, spectral alterations were more pronounced, with strong peaks at 541, 713, 1031, 1125, 1442, and 1657 cm ⁻ ¹, indicating molecular signatures characteristic of malignant transformation (Fig 3D). When we compared unique peaks of each group, we found that the normal group had unique peaks at 637 and 1207 cm ⁻ ¹. In the inflammation group, we found specific peaks at 1235 and 1343 cm ⁻ ¹. However, Pre-CA and CCA groups resembled each other in peak patterns.

### PCA-based follow-up of Raman signal changes

Principal component analysis (PCA) was used to reduce the dimensionality of the raw spectral data, which consisted of 1500 variables (wavenumbers). We retained the first 20 principal components (PCs) for all subsequent classification models based on two key considerations. First, these 20 PCs accounted for most of the total variance in the data, which ensured that important spectral information was not lost. Second, given the high degree of similarity observed between the Raman spectra of the different pathological classes, retaining a larger number of PCs than is typical was a deliberate strategy. This approach was chosen to preserve the more subtle, lower-variance components that could contain critical information for discriminating between the groups.

**Fig 4:**
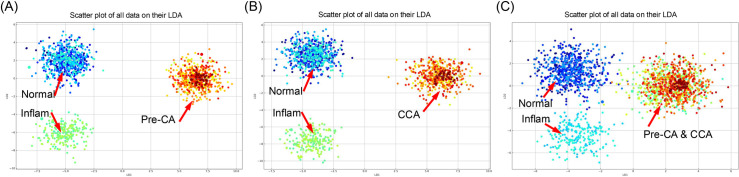
PCA-LDA analysis in different stages of CCA evolution. Within the four-class model, (A) the precancerous (Pre-CA) and **(B)** CCA groups clustered closely together, but the normal (blue) and inflammatory (cyan) samples formed separate, non-overlapping clusters. **(C)** Illustrates the three-class model that was investigated, which combined Pre-CA and CCA into a single category.

The PCA-LDA analysis of serum SERS spectra effectively distinguished between different stages of CCA progression, shown in Fig. 4. In the four-class model, the normal (blue) and inflammation (cyan) samples formed distinct, non-overlapping clusters, while the precancerous (Pre-CA) (Fig 4A) and CCA groups (Fig 4B) clustered closely together, suggesting shared spectral features during cancer progression. Despite this proximity, the model successfully differentiated among all four groups, highlighting the biochemical changes associated with the development of CCA. To improve classification accuracy, a three-class model was explored, grouping pre-CA with CCA in one category, as shown in Fig 4C. This approach maintained the clear separation between the normal and inflammatory groups, while still distinguishing the cancer-related category (Pre-CA/CCA) from non-cancerous states. The three-class PCA-LDA model demonstrated more defined clustering patterns, suggesting that reducing classification complexity improves spectral discrimination. The integration of PCA-LDA with SERS spectral data demonstrated high discriminatory performance, with the three-class model showing potential advantages in classification accuracy.

### Diagnostic performance using SERS and machine-learning algorithms

The classification performance was evaluated for both four-class (normal, inflammation, precancerous, CCA) and three-class (normal, inflammation, CCA/precancerous combined) models using train-test split and leave-one-out cross-validation (LOOCV) approaches. In the four-class model, the train-test split achieved an overall accuracy of 62%, with a high recall (0.84) for normal samples. It means that the model effectively identifies target samples and minimizes missed detections. However, lower performance was observed for inflammation (recall = 0.07, F1-score = 0.11), indicating challenges in distinguishing inflammation from other stages. The precancerous and CCA groups had moderate classification performance, with F1-scores of 0.56 and 0.65, respectively. The LOOCV model demonstrated a reduced overall accuracy (47%), with high recall for normal samples (0.87), but poor recall for inflammation (0.00) and precancerous lesions (0.07), indicating variable sensitivity in sample classification (Table 3).

**Table 3:** Accuracy values of the four-class model (normal, inflammation, precancerous, CCA).

Four-class model and their performance	Train-Test Split: Classification Report					LOOCV: Classification Report				
Precision	Recall	F1-score	Support	Specificity	Precision	Recall	F1-score	Support	Specificity
Group 1-Normal	0.61	0.84	0.71	231	0.95	0.52	0.87	0.65	15	0.65
Group 2 -Inflam	0.40	0.07	0.11	61	0.99	0.00	0.00	0.00	5	1.00
Group 3-Pre-Ca	0.61	0.51	0.56	210	0.87	0.17	0.07	0.10	14	0.86
Group 4-CCA	0.65	0.65	0.65	220	0.85	0.53	0.60	0.56	15	0.77
Test Accuracy			0.62	722				0.47	49	
Macro average	0.57	0.52	0.51	722		0.30	0.38	0.33	49	
Weighted average	0.61	0.62	0.60	722		0.37	0.47	0.40	49	

Precision: measures the accuracy of positive predictions; Recall (sensitivity): measures the model’s ability to find all the positive instances; F1-score: interpreted as a harmonic mean of the precision and recall, where an F1 score reaches its best value at 1 and worst score at 0; Support: number of samples in each class.

In contrast, the three-class model, which combined precancerous and CCA groups, exhibited improved classification performance. The train-test split model achieved 80% accuracy, with high precision (0.93) and recall (0.93) for CCA. The LOOCV model had an accuracy of 68%, demonstrating improved robustness compared to the four-class model. However, the inflammation group remained poorly classified (recall = 0.00 in both models), suggesting spectral similarities with other groups (Table 4).

**Table 4:** Accuracy values of the three-class model (normal, inflammation, and combined precancerous and CCA).

Three-class model and their performance	LOOCV: Classification Report For Group 3 as Pre-CA					LOOCV: Classification Report For Group 3 as CCA				
Precision	Recall	F1-score	Support	Specificity	Precision	Recall	F1-score	Support	Specificity
Group 1-Normal	0.60	0.80	0.69	15	0.60	0.70	0.93	0.80	15	0.70
Group 2 -Inflam	0.00	0.00	0.00	5	1.00	0.00	0.00	0.00	5	1.00
Group 3	0.79	0.79	0.79	14	0.85	0.93	0.93	0.93	15	0.95
Accuracy			0.68	34				0.80	35	
Macro average	0.46	0.53	0.49	34		0.54	0.62	0.58	35	
Weighted average	0.59	0.68	0.68	34		0.70	0.80	0.74	35	

To assess model generalizability and diagnose potential overfitting, we systematically compared the training and validation performance across all models. For the four-class classification, a standard train-test split yielded identical training and validation accuracies of 0.62, suggesting a well-generalized model for that specific data partition. In the more rigorous LOOCV, the mean training accuracy was 0.57 ± 0.01, while the mean validation accuracy was 0.47. This modest drop in performance on unseen individual subjects is expected and provides a more realistic estimate of the model’s predictive power. The improved robustness of the three-class models was evident in this analysis. The Normal-Inflam-PreCA model showed close agreement between training (0.73 ± 0.01) and validation (0.68) accuracies. This trend was even stronger in the Normal-Inflam-CCA model, which achieved a mean training accuracy of 0.81 ± 0.01 and a nearly identical validation accuracy of 0.80. Collectively, the consistent agreement between training and validation scores—particularly in the high-performing three-class models—provides strong evidence that our models are robust, well-generalized, and not subject to significant overfitting.

Moreover, to determine if the classification outcomes of our two validation approaches (train-test split vs. LOOCV) were statistically different, we employed McNemar’s test. Since the three-class LOOCV models were identified as optimal, we focused the comparison on their performance for the Pre-CA and CCA groups. For the Pre-CA group, the test revealed no significant difference between the two methods’ predictions (χ² = 0, *p* = 1.000), indicating that both validation strategies led to identical classification outcomes for this cohort. In contrast, for the CCA group, a statistically significant difference was found (χ² = 4.167, *p* = 0.041). This result indicates that the two validation approaches produced discordant predictions for the CCA samples, highlighting that the choice of validation strategy significantly impacts the classification results for this specific group. Two-by-two raw data for 3-class and 4-class models by McNemar’s test are shown in S3 Table.

## Discussion

In this study, we developed a minimally invasive diagnostic approach using surface-enhanced Raman spectroscopy (SERS) combined with machine learning to distinguish different stages of cholangiocarcinoma (CCA) development in a hamster model. Our findings demonstrated the effectiveness of SERS in detecting biochemical changes associated with CCA progression, with notable differences in Raman spectral features across normal, inflammation, precancerous (Pre-CA), and CCA groups. The integration of SERS data with principal component analysis-linear discriminant analysis (PCA-LDA) and other machine-learning algorithms yielded high discriminatory performance, establishing the foundation for a rapid, potentially minimally invasive SERS-based diagnostic platform. This platform holds special promise for early-stage CCA detection and monitoring, particularly for targeted screening of individuals with evolving risk factors or in endemic areas where early detection is crucial even without overt symptoms.

Various techniques are used for CCA diagnosis; the definitive diagnosis is made after histopathological confirmation [29]. In the present study, we classified the stage of CCA development based on histopathological changes into four categories: normal, inflammatory, precancerous (Pre-CA), and CCA. In addition to our earlier finding that the increase in fibrosis accompanies CCA development [30], fibrosis alone, without tumor development and absent fluke involvement, was excluded from this study. Our histopathological classification findings are consistent with known processes of CCA development (17, 18), possibly via oval cell hyperplasia and aberrant ductular proliferation [31]. Moreover, the expression of bipotential progenitor stem cells, leading to the expression of CK-19 and alpha-fetoprotein (AFP) markers, was measured to support the histopathological findings. Our experiment mimics routine practice and guidelines that a definitive diagnosis of CCA can be made after histopathological examination of the primary tumor [32]. Relevantly, for CCA screening by ultrasound in the community [33], searching for the risk condition of fluke-associated CCA is required for histopathological confirmation [4].

Based on histopathological features (normal, inflammatory, pre-CA, and CCA), we used Raman spectroscopy analysis to search for a unique biomarker for CCA diagnosis. Raman spectroscopy revealed distinct biochemical signatures for each tissue group, progressing from a healthy to a cancerous state. The baseline metabolism of normal samples was characterized by peaks at 537, 637, 854, 1002, 1449, and 1660 cm ⁻ ¹, reflecting the standard chemical information of cellular lipids, proteins, and nucleic acids [34,35]. In the early stages of disease, inflammation-induced spectral alterations, with characteristic shifts appearing at 538, 758, 854, 928, 1002, 1343, 1449, and 1666 cm ⁻ ¹. Alterations of these spectra may reflect early inflammatory changes [36]. As the tissue advanced to a precancerous state, a different set of peaks emerged at 542, 715, 1003, 1441, and 1657 cm ⁻ ¹, indicating the initial molecular changes associated with malignancy [37]. Finally, fully developed CCA was characterized by significant peaks at 541, 713, 1031, 1125, 1442, and 1657 cm ⁻ ¹, highlighting their potential for diagnosis, similar to approaches used for breast cancer [38]. These progressive spectral alterations correspond to modifications in nucleic acids, proteins, and lipids that occur during malignant transformation [38–40]. The clear distinction in Raman peaks between precancerous and cancerous tissues suggests this technique could be a valuable tool for the early diagnosis of CCA, similar to its application in other cancers like breast and cervical cancer [38,41].

The integration of SERS spectral data with PCA-LDA and machine learning demonstrated high classification efficiency for CCA staging. The four-class model (normal, inflammation, Pre-CA, CCA) achieved a train-test accuracy of 62% but struggled to distinguish inflammation from normal samples (recall = 0.07, F1-score = 0.11) due to spectral overlaps. In contrast, the three-class model (normal, inflammation, Pre-CA/CCA combined) improved performance, achieving an accuracy of 80% (train-test split) and 68% (LOOCV). This improvement is likely due to the biochemical similarities between Pre-CA and CCA. In the four-class classification, the Pre-CA and CCA groups exhibited overlapping spectral features, making it challenging for the model to differentiate between these two stages with high confidence. By merging Pre-CA and CCA into a single category, the model reduced classification complexity, thereby enhancing its ability to distinguish between cancer-related states and non-cancerous conditions. The high precision (0.93) and recall (0.93) for Pre-CA/CCA classification demonstrate the robust potential of SERS-based machine-learning models for identifying this category.

Only male hamsters were employed in this study to reduce the possibility of female hormone cycles confusing the results. The generalizability of our findings to both sexes may be limited by this restriction. Further research involving both sexes will be required to completely comprehend any effects that are particular to one sex. Additionally, a primary limitation of this study was the small sample size of the inflammatory (Inflam) group, which introduced classification bias and reduced the statistical power of the machine learning models. This imbalance led to diminished performance, evidenced by markedly lower precision and recall for the Inflam group, particularly under LOOCV testing. The challenge was compounded because the biological specimens represent a continuous spectrum of biochemical variation; limited samples can disproportionately amplify this inherent variability and hinder the model’s ability to generalize. The small sample size resulted from a limited initial cohort combined with animal attrition during the experiment. While this factor reduced accuracy, the study was intentionally designed as a proof-of-concept. The results successfully demonstrate the feasibility of using serum-based SERS with machine learning to distinguish between disease states, while strongly highlighting that future investigations will require larger, more balanced datasets to improve model robustness and classification accuracy.

## Conclusion

This study demonstrates the potential of SERS combined with machine learning as a rapid, cost-effective diagnostic tool for early-stage CCA detection, with identified stage-specific Raman peaks offering insights into biochemical progression. The model’s low accuracy (>67%) and AUC of ROC (>0.67) for precancerous lesions, despite its promising sensitivity (93%) and specificity (95%), indicate that more optimization is necessary to improve the effectiveness of early screening. The capacity of the model to distinguish between high-grade dysplasia and early cancer, external validation using separate human clinical samples, and connecting SERS+ML classifications to underlying biochemical alterations to enhance scientific relevance are crucial future steps. To improve overall diagnosis accuracy and utility in CCA screening programs, future research should further increase sample size, integrate SERS with other diagnostic modalities, and use sophisticated machine-learning approaches like deep learning.

## Supporting information

S1 Table Raw data of histopathological findings by the investigator 1. (DOCX)

S2 Table Raw data of histopathological findings by the investigator 2. (DOCX)

S3 Table Two-by-two raw data for 3-class and 4-class models by McNemar’s test. (DOCX)
