# Supplementary material for: Minimally invasive detection of early-stage opisthorchiasis-associated cholangiocarcinoma using label-free surface-enhanced Raman spectroscopy (SERS) of hamster serum
Source: PLoS One. 2025 Oct 27;20(10):e0334916. doi: 10.1371/journal.pone.0334916 (PMC12558545; doi:10.1371/journal.pone.0334916)
Supplement: S2 Table — (DOCX) [file pone.0334916.s002.docx]

**S2 Table. Raw data of histopathological findings by the investigator 2.**

| Duration | Case number | Perihilar chronic infiammatory cell foci | Peripheral chronic inflammatory cell foci | Cholangiofibrosis | Cholangiofibrosis | Cholangiocarcinoma | Group |
| --- | --- | --- | --- | --- | --- | --- | --- |
| 3M | 4 | 1 | 2 | 2 | 1 | 2 | CCA |
| 3M | 5 | 1 | 2 | 1 | 1 | 1 | CCA |
| 4M | 7 | 2 | 3 | 2 | 0 | 1 | CCA |
| 5M | 1 | 1 | 2 | 2 | 2 | 1 | CCA |
| 5M | 4 | 1 | 2 | 3 | 1 | 1 | CCA |
| 5M | 7 | 1 | 1 | 2 | 2 | 2 | CCA |
| 5M | 10 | 1 | 2 | 2 | 1 | 1 | CCA |
| 5M | 3 | 2 | 3 | 2 | 1 | 2 | CCA |
| 5M | 6 | 1 | 2 | 4 | 2 | 1 | CCA |
| 5M | 7 | 1 | 3 | 2 | 1 | 2 | CCA |
| 5M | 8 | 1 | 2 | 2 | 1 | 1 | CCA |
| 5M | 9 | 1 | 2 | 2 | 0 | 1 | CCA |
| 4M | 1 | 1 | 2 | 2 | 1 | 1 | CCA |
| 4M | 2 | 1 | 2 | 2 | 1 | 2 | CCA |
| 4M | 4 | 1 | 1 | 3 | 1 | 1 | CCA |
| 1M | 1 | 1 | 1 | 0 | 0 | 0 | Inflammation |
| 1M | 2 | 1 | 1 | 0 | 0 | 0 | Inflammation |
| 1M | 3 | 1 | 3 | 0 | 0 | 0 | Inflammation |
| 1M | 4 | 1 | 2 | 0 | 0 | 0 | Inflammation |
| 1M | 5 | 1 | 1 | 0 | 0 | 0 | Inflammation |
| 3M | 1 | 1 | 2 | 2 | 0 | 0 | Pre-CA |
| 3M | 2 | 1 | 2 | 2 | 1 | 0 | Pre-CA |
| 3M | 3 | 1 | 2 | 2 | 1 | 0 | Pre-CA |
| 4M | 3 | 1 | 1 | 3 | 0 | 0 | Pre-CA |
| 4M | 5 | 1 | 3 | 2 | 1 | 0 | Pre-CA |
| 4M | 6 | 1 | 2 | 4 | 2 | 0 | Pre-CA |
| 4M | 8 | 2 | 3 | 2 | 1 | 0 | Pre-CA |
| 4M | 9 | 2 | 3 | 3 | 1 | 0 | Pre-CA |
| 5M | 2 | 2 | 2 | 3 | 0 | 0 | Pre-CA |
| 5M | 3 | 1 | 3 | 2 | 1 | 0 | Pre-CA |
| 5M | 5 | 1 | 3 | 3 | 1 | 0 | Pre-CA |
| 5M | 1 | 1 | 2 | 4 | 1 | 0 | Pre-CA |
| 5M | 2 | 1 | 2 | 4 | 1 | 0 | Pre-CA |
| 5M | 4 | 1 | 2 | 4 | 2 | 0 | Pre-CA |
| 5M | 5 | 1 | 1 | 3 | 0 | 0 | Pre-CA |

**Summary of S2 Table**

|  | Inflammation | Cholangiofibrosis | Cholangiofibrosis | Tumor |
| --- | --- | --- | --- | --- |
| CCA | 2.066666667 | 2.2 | 1.066666667 | 1.333333333 |
| Pre-cancerous | 2.2 | 2.866666667 | 0.866666667 | 0 |
| Inflammation | 1.6 | 0 | 0 | 0 |
