# Supplementary material for: Minimally invasive detection of early-stage opisthorchiasis-associated cholangiocarcinoma using label-free surface-enhanced Raman spectroscopy (SERS) of hamster serum
Source: PLoS One. 2025 Oct 27;20(10):e0334916. doi: 10.1371/journal.pone.0334916 (PMC12558545; doi:10.1371/journal.pone.0334916)
Supplement: S3 Table — (DOCX) [file pone.0334916.s003.docx]

**S3 Table. Two-by-two raw data for 3-class and 4-class models by McNemar's test.**

| With Pre-CA Group (n=34) | 3-class model correct | 3-class model incorrect |
| --- | --- | --- |
| 4-class model correct | 22 | 0 |
| 4-class model incorrect | 1 | 8 |

| With CCA Group (n=35) | 3-class model correct | 3-class model incorrect |
| --- | --- | --- |
| 4-class model correct | 22 | 0 |
| 4-class model incorrect | 6 | 8 |
